# Biofilm matrix regulation by *Candida glabrata* Zap1 under acidic conditions: transcriptomic and proteomic analyses

**DOI:** 10.1128/spectrum.01201-24

**Published:** 2024-11-04

**Authors:** Bruna Gonçalves, Diana Priscila Pires, Liliana Fernandes, Miguel Pacheco, Tiago Ferreira, Hugo Osório, Ana Raquel Soares, Mariana Henriques, Sónia Silva

**Affiliations:** 1 Centre of Biological Engineering (CEB), University of Minho, Braga, Portugal; 2 LABBELS—Associate Laboratory, Braga/Guimarães, Portugal; 3 Informatics Department, University of Minho, Braga, Portugal; 4 i3S—Institute for Research and Innovation in Health, University of Porto, Porto, Portugal; 5 Ipatimup—Institute of Molecular Pathology and Immunology of the University of Porto, University of Porto, Porto, Portugal; 6 Department of Pathology, Faculty of Medicine, University of Porto, Porto, Portugal; 7 Institute of Biomedicine, Department of Medical Sciences, University of Aveiro, Aveiro, Portugal; 8 National Institute for Agrarian and Veterinary Research, Vairão, Vila do Conde, Portugal; Universidade do Minho, Braga, Portugal

**Keywords:** *Candida *spp., biofilm, transcriptome, proteome, vaginal environment, Zap1 targets

## Abstract

The vaginal acidic environment potentiates the formation of *Candida glabrata* biofilms, leading to complicated and recurrent infections. Importantly, the production of matrix is known to contribute to the recalcitrant features of *Candida* biofilms. In this study, we reveal that Zap1 regulates the matrix of *C. glabrata* acidic biofilms and analyzed the modulation of their transcriptome (by microarrays) and matrix proteome (by LC-MS/MS) by Zap1. For that, the deletion mutant *zap1*Δ and its complemented strain *zap1*Δ::*ZAP1* were constructed, and their biofilms were developed at pH 4 (adjusted with lactic acid). The results revealed that Zap1 is a negative regulator of the total amount of protein and carbohydrate in the biofilm matrix. Accordingly, various genes and matrix proteins with predicted functions in the regulation of carbohydrate metabolism, sugar binding, sugar transport, and adhesion (including Epa family) were repressed by Zap1. Nevertheless, the results also suggested that Zap1 is essential to the delivery and organization of some matrix components. Indeed, Zap1 was required for the secretion of 122 proteins to the matrix and induced the expression of 557 genes, including various targets involved in glucan metabolism. Additionally, Zap1 induced targets with roles in virulence, resistance to antifungals, and host immunity evasion, including yapsins, *ERG* family, and moonlighting proteins. Zap1 was also required for the secretion of acidic-specific matrix proteins, indicating a contribution to the response to the acidic environment. Overall, this study demonstrates that Zap1 is a relevant regulator of the biofilm matrix, contributing to a better understanding of *C. glabrata* acidic biofilms.

**IMPORTANCE:**

The rising prevalence of vulvovaginal candidiasis (VVC) and the increasing presence of *Candida* spp. with aggressive virulence features and low susceptibility to common antifungals, particularly *Candida glabrata*, have resulted in more severe, prolonged, and recurrent cases of VVC, with significant implications for patients. This research offers valuable insights into the molecular changes that contribute to the formation of *C. glabrata* biofilms in the acidic vaginal environment, representing a significant advancement in the understanding of *C. glabrata*’s virulence. Notably, this study identified Zap1 as a critical regulator of *C. glabrata* biofilm matrix, with additional potential roles in adhesion, antifungal resistance, evasion of host immunity, and response to acidic conditions, making it a promising target for new therapeutic approaches. Importantly, Zap1 is the first regulator of the biofilm matrix to be identified in *C. glabrata*, and the elucidation of its targets (including genes and matrix proteins) lays a strong foundation for future research.

## INTRODUCTION

*Candida* species are opportunistic pathogens responsible for infections in several anatomical sites (1–3), where they have to adapt to different environmental conditions, including various pH levels (4). The acidic pH of the vaginal tract, which is mainly prompted by the lactic acid, does not inhibit *Candida* growth in contrast with its effect on most vaginal pathogens (5, 6). *Candida glabrata* is the second most common causative agent of vulvovaginal candidiasis (VVC), following *Candida albicans*, and is one of the most clinically relevant species due to its intrinsic high resistance to antifungal agents and its aggressive virulence factors, including highly recalcitrant biofilms (3, 7). We have recently showed that vaginal acidic pH potentiates the formation of *C. glabrata* biofilms and modulates the amount of carbohydrate and protein secreted into the matrix (8). Matrix carbohydrates have a potential role in *C. glabrata* biofilm antifungal resistance (9–11). The role of matrix proteins is unclear but according to the proteomic profile of *C. glabrata* biofilm matrix recently revealed by us, potential roles in the delivery/organization of matrix carbohydrates, virulence, and adhesion may be suggested (12). In addition, we found that several matrix proteins were specifically secreted into *C. glabrata* biofilm matrix under acidic conditions (12).

Biofilm matrix composition and its regulation have been suggested to have a greater impact on biofilm resistance features than the limitation of harmful substances diffusion by the matrix structure (13). Although several studies have focused on the genetic regulation of *C. albicans* biofilm matrix (14–18), only a few have studied the matrix regulation in *C. glabrata* (11, 19). An interesting biofilm matrix regulator reported in *C. albicans* is the transcription factor Zap1, which was reported to be a regulator of glucan delivery to the biofilm matrix (15). *Candida albicans* Zap1 possesses at least 525 target genes already identified, according to PathoYeastract (20), and a study of the transcriptome modulation by Zap1 has been performed with *C. albicans* biofilms (15). However, in *C. glabrata*, the role of Zap1 in biofilm matrix production is unknown and no target genes have yet been revealed for Zap1. As such, this study aimed to reveal and analyze *C. glabrata* biofilm matrix regulation by the transcription factor Zap1, under acidic conditions (settled with lactic acid). For that, its role on biofilm phenotypic features, including the amount of matrix components, transcriptome, and matrix proteome, was analyzed using a mutant strain lacking the *ZAP1* gene. This study is essential to clarify the role of Zap1 in the development of *C. glabrata* biofilms, contributing to a better understanding of matrix regulation in vaginal niche conditions and, thus, deepening the knowledge about this clinically relevant species.

## MATERIALS AND METHODS

### Construction of mutant strains

The reference strain *C. glabrata* ATCC 2001 was genetically manipulated in order to obtain two strains: a knockout mutant lacking the *ZAP1* gene (*zap1*Δ) and its complemented strain, containing the reintegrated gene (*zap1*Δ::*ZAP1*). To construct the mutant strains, the *SAT1*-flipping strategy was applied as previously described for *C. albicans* (21, 22), with some modifications to delete the *ZAP1* gene in *C. glabrata*.

#### Assembly of the deletion cassette

For the construction of the deletion cassette, *C. glabrata* ATCC 2001 was grown on Yeast Peptone Dextrose (YPD; Sigma-Aldrich, St Louis, MO, USA) agar plates for 48 h and then in YPD medium incubated overnight, under agitation at 30°C. Next, the genomic DNA was extracted from overnight grown cells using a yeast DNA extraction kit (Zymo Research, Irvine, CA, USA) according to the manufacturer’s instructions. The DNA was used as a template for the PCR amplification of sequences of approximately 500 base pairs (bp) length located upstream and downstream of *C. glabrata ZAP1* gene, with primers specifically designed for these sequences (Table S1) and using the Phusion High-Fidelity DNA Polymerase (Thermo Scientific, Bremen, Germany). *Apa* I and *Xho* restriction sites were included in the primers amplifying the upstream sequence of *ZAP1* gene, and *Sac* II and *Sac* I restriction sites were added to those amplifying the downstream sequence of *ZAP1* gene (Table S1; Fig. S1a). Amplicons were cleaned (DNA Clean & Concentrator-5; Zymo Research, Irvine, CA, USA) and digested with the respective restriction enzymes (Thermo Scientific, Bremen, Germany). A plasmid containing the *SAT1* flipper cassette (pSFS2) was digested with the same restriction enzymes, leading to the excision of the *SAT1* flipper deletion cassette from the remaining vector backbone (Fig. S1a). After the digestions, the DNA fragments obtained were separated by agarose gel electrophoresis, excised from the gel, and purified using a gel DNA recovery kit (Zymo Research, Irvine, CA, USA) according to the manufacturer’s instructions. Then, a quadruple ligation was performed with the digested vector backbone, the cassette, and the upstream and downstream fragments (obtained by PCR), by incubating them with T4-DNA ligase (Thermo Scientific, Bremen, Germany) overnight at 16°C. Due to the compatible extremities obtained after the digestions, this process generated a plasmid construct formed by the *SAT1* flipper cassette flanked by the upstream and downstream *ZAP1* sequences (Fig. S1a).

The ligation was used to transform chemically competent *Escherichia coli* TOP10 cells by heat shock (incubation for 30 min on ice, followed by 45 s at a 42°C and 2 min on ice). After transformation, the cells were recovered with Super Optimal broth with Catabolite repression medium (SOC; Sigma-Aldrich, St Louis, MO, USA) and incubated at 37°C under shaking for 1 h. Transformed cells were plated onto Lysogeny Broth (LB; Sigma-Aldrich, St Louis, MO, USA), agar plates supplemented with 100 µg/mL ampicillin to select positive transformants that incorporated the plasmid. The resulting colonies were screened by colony PCR (primers presented in Table S1), and the positive ones were used for plasmid extraction using a plasmid miniprep kit (Zymo Research, Irvine, CA, USA) according to the manufacturer’s instructions. The correct assembly of the plasmid was further confirmed by Sanger sequencing (Eurofins Genomics). Then, the complete deletion cassette was excised from the plasmid, by digesting it with *Apa* I and *Sac* I enzymes. The obtained fragments were separated by agarose gel electrophoresis, and the deletion cassette was extracted from the gel, purified with the gel DNA recovery kit (Zymo Research, Irvine, CA, USA), and transformed into *C. glabrata* cells, described as follows.

#### Transformation of C. glabrata cells

*Candida glabrata* ATCC 2001 electrocompetent cells were prepared according to a previously described protocol (21). One hundred micro liters of the electrocompetent cells were then mixed with the purified DNA fragment containing the deletion cassette and incubated for 5 min on ice, followed by electroporation at 1.8 kV. This process allowed the cassette to integrate into *C. glabrata* genome by replacing *ZAP1* gene through the homologous recombination of the flanking sequences (Fig. S1b). After electroporation, cells were resuspended in a YPD medium and incubated for 4 h at 30°C under agitation (120 rev/min). Next, the suspensions were spread on YPD agar plates supplemented with nourseothricin (200 µg/l), and incubated for 48 h at 30°C, to select the nourseothricin-resistant transformants that have integrated the cassette. Colony PCR was performed to confirm the swap of *ZAP1* gene by the *SAT1* flipper cassette. The selected transformants were then incubated overnight in Yeast Peptone Maltose (YPM) medium at 30°C under shaking in order to perform a maltose-induced excision of the cassette. Finally, the cells were spread on YPD agar plates containing a low concentration of nourseothricin (20 µg/l) (Thermo Scientific, Bremen, Germany) and incubated for 48 h at 30°C. Cells that have lost the *SAT1* flipper cassette became nourseothricin-sensitive and appeared as small colonies, whereas the cells that have retained the cassette formed large colonies. To confirm that small colonies corresponded to the knockout mutant desired (*zap1*Δ), the absence of the *ZAP1* gene and also the *SAT1* cassette were confirmed by PCR amplification (Fig. S2) and Sanger sequencing.

#### Construction of the complemented strain

In order to complement the knockout mutant strain by re-integrating the deleted *ZAP1* gene, all the procedures previously described were similarly performed but using the knockout mutant (*zap1*Δ) for the transformation step instead of *C. glabrata* ATCC 2001. Additionally, the construction of the deletion cassette was adapted to also include the *ZAP1* gene. The obtained complemented strain (*zap1*Δ::*ZAP1*) was confirmed by PCR amplification (Fig. S2) and Sanger sequencing (the primers designed for the assembly of the complemented deletion cassette and the confirmation of the construct are presented in Table S1).

### Strains and initial growth conditions

In this study, the mutant strains *C. glabrata zap1*Δ and *zap1*Δ::*ZAP1*, constructed as described above, and the reference strain *C. glabrata* ATCC 2001 were used for various microbiologic and molecular analyses. These strains were initially grown in Sabouraud dextrose agar (SDA; Merck, Darmstadt, Germany) at 37°C, for 48 h, followed by 18 h in Sabouraud dextrose broth (SDB; Merck, Darmstadt, Germany) under 120 rev/min. The cells were washed with phosphate-buffered saline 1× (PBS; pH 7), and the pellet was resuspended in Roswell Park Memorial Institute (RPMI; Sigma-Aldrich, St Louis, MO, USA) medium settled to pH 4 (with lactic acid) and supplemented with 2% of glucose. Importantly, zinc solubility is dependent on the pH, and its dissolution is faster in acidic than neutral medium (23). As such, it is not expected a limitation in zinc availability. Then, the cellular density of the inoculums was adjusted to 1 × 10^5^ cells/mL, using a Neubauer haemocytometer for cells counting (Marienfeld, Lauda-Königshofen, Germany). Three independent inoculums were prepared for each experiment.

### Planktonic growth and analysis

For the planktonic growth, 25 mL of the inoculums (cell cultures with adjusted cell concentration) prepared in RPMI at pH 4, as described above, were incubated for 24 h at 37°C under agitation (120 rev/min). The optical density of the suspensions (at 690 nm) was measured over time using a microtiter plate reader (Bio-Tek Synergy HT, Izasa, Winooski, VT, USA) (8). Additionally, the number of cultivable cells after 24 h of growth was determined by colony-forming units (CFU) counting methodology, as previously described (8). Briefly, a serial of 10-fold dilutions of the planktonic suspensions were prepared in PBS, plated on SDA, and incubated for 24 h at 37°C. After incubation, the number of CFUs was counted, and the results were presented as Log CFUs/mL.

### Biofilm formation and analysis

For the development of biofilms, the inoculums were placed into wells of 96-wells polystyrene microtiter plates (Orange Scientific, Braine-l`Alleud, Belgium) (200 µL per well) and incubated at 37°C under agitation (120 rev/min) for 24 h (24). After incubation, biofilms were washed with PBS to remove non-adherent cells and then analyzed.

The biofilms’ total biomass was analyzed by Crystal Violet (CV) staining methodology (24). Briefly, biofilms were stained with CV (1%, vol/vol) for 5 min after being fixed with methanol for 15 min. Then, the biofilms were washed with water, and the dye was released with acetic acid (33%, vol/vol). The absorbance of the solutions was read at 570 nm on a microtiter plate reader (Bio-Tek Synergy HT, Izasa, Winooski, Vermont). Additionally, the number of cultivable cells in the biofilms was analyzed by CFU counting methodology. For that, formed biofilms were scraped from the wells with PBS, and the suspensions were analyzed as described above for planktonic cells. The results of these analyses were presented per unit area of biofilm.

Formed biofilms were also visualized with a confocal laser scanning microscope (CLSM; Olympus BX61, Model FluoView 1000, Portugal), using the excitation line 405 and the emission filters BA 430–470 (blue channel) (8). For that, biofilms were firstly stained with Calcofluor white (1%, vol/vol; Sigma-Aldrich, St Louis, MO, USA) for 10 min in the dark at room temperature. The images were acquired with the program FV10-ASW 4.2 (Olympus, Portugal). The biofilm thickness was analyzed in three areas of each image, and the median thickness value was calculated for each replicate.

### Planktonic supernatant and biofilm matrix composition analysis

For the analysis of the planktonic supernatants and biofilm matrices*, C. glabrata* cells were grown as described above. Biofilms were separated from their matrices by sonication for 30 s at 30 W (Ultrasonic Processor, Cole-Parmer, Vernon Hills, IL, USA) followed by centrifugation (24). The planktonic cells were also separated from their supernatants by centrifugation. The supernatant and matrix-containing suspensions were filtered through a 0.2-µm nitrocellulose filter. Then, the amount of protein, total carbohydrate, and (1,3)-β-d-glucan were measured, using the BCA Kit, phenol-sulfuric method, and Glucatell (1,3)-β-d-Glucan Detection Reagent kit, respectively, as previously described in detail (8). The results of the components of planktonic supernatants were presented per milliliter of supernatant, and those of the matrices per gram of the biofilm cells’ dry weight. The biofilm dry weight was achieved by drying the biofilm cells separated from the matrices, at 37°C, until a constant weight was obtained. Three biological replicates and three technical replicates of each strain in each condition were included in these analyses.

### Proteomic analysis of the biofilm matrix

The proteins of *zap1*Δ and *zap1*Δ::*ZAP1* biofilm matrices were identified by nano liquid chromatography-tandem mass spectrometry (LC-MS/MS), using an Ultimate 3000 liquid chromatography system coupled to a Q-Exactive Hybrid Quadrupole-Orbitrap mass spectrometer (Thermo Scientific, Bremen, Germany), as previously described in detail (12). The experimental setups used to form *zap1*Δ and *zap1*Δ::*ZAP1* biofilms and to extract their matrices were the same as described above. Briefly, matrix-containing samples were digested with trypsin/Lys-C mix overnight at 37°C in triethylamonium bicarbonate (TEAB) after being reduced and alkylated with tris(2-carboxyethyl)phosphine (TCEP), carboxylic acid amide (CAA), and urea/TEAB. The digestion was stopped with trifluoroacetic acid (TFA), the peptides were recovered by centrifugation, and the peptide samples were cleaned up and concentrated by chromatography before the nano LC-MS/MS analysis. Data acquisition was controlled by Xcalibur 4.0 and Tune 2.8 software (Thermo Scientific).

The raw data of LC–MS/MS analysis were processed using Proteome Discoverer 2.2.0.388 software (Thermo Scientific) and searched against the UniProt database (25) for the taxonomic selection *C. glabrata*. The tryptic peptides were identified with the Sequest HT search engine, allowing a maximum missing cleavage of 2. The tolerance of ion mass for precursor and fragmented ions was set to 10 ppm and 0.02 Da, respectively. The settings of the processing node Percolator were decoy database search target FDR 1% and maximum delta Cn 0.05. Two biofilm matrix replicates of each strain were used for the analyses, and only proteins found in both replicates were selected for further analyses.

### Transcriptomic analysis

The transcriptomic analysis of *zap1*Δ and *zap1*Δ::*ZAP1* strains was performed using species-specific DNA microarrays (26). For this, dual-channel microarray analyses (control vs test) of cells of both strains grown in planktonic (control) vs biofilm (test) lifestyles were performed. The experimental setups used to grow *zap1*Δ and *zap1*Δ::*ZAP1* cells were the same as described above. Cell suspensions with planktonic and biofilm cells were centrifuged, and the pellets were used for RNA extraction and microarrays analysis.

#### RNA extraction

For the RNA extraction from the cells, the RiboPure—Yeast Kit (Life Technologies, Carlsbad, CA, USA) was used according to the manufacturer’s guidelines. Spectrophotometry was used to determine the concentration and purity of the extracted RNA, and its integrity was analyzed with an Agilent 2100 Bioanalyzer with an RNA 6000 Nano Assay (Agilent Technologies, Santa Clara, CA, USA). An RNA integrity number (RIN) >7 was settled to select the samples to be used for the microarray analysis.

#### Microarrays

The Agilent protocol for two-color Microarray-Based Gene Expression Analysis Low Input Quick Amp Labeling v6.9 (Agilent Technologies) was followed to perform cDNA synthesis, hybridization, and scanning, as previously described (27). Briefly, an Agilent T7 Promoter Primer and T7 RNA polymerase Blend (Agilent Technologies, Cat. 5190–2305) were used to synthesize labeled cDNA (with Cyanine 3-CTP and cyanine 5-CTP) from the extracted RNA. Then, labeled cDNA was hybridized in Agilent microarray slides and placed in Agilent gasket slides in a rotating oven at 65°C for 17 h. Microarray slides were washed following the manufacturer’s protocol and scanned in an Agilent G2565AA microarrays scanner, and the probe signal values were extracted using Agilent Feature Extraction Software. Three biological replicates and three technical replicates were performed in each analysis. Data normalization was accomplished by median centering of signal distribution with Biometric Research Branch BRB-Array tools v3.4.o software. The LIMMA package in MeV software 36 (MultiExperiment Viewer 4.8.0) was used for final statistical analysis, assuming a cut-off *P*-value of 0.01.

### Measurement of transcripts by quantitative real-time PCR

The expression level of *ZAP1* in *C. glabrata* ATCC 2001 cells grown in planktonic and biofilm modes at pH 4 was assessed using quantitative real-time PCR (qRT-PCR). The experimental setups used to cultivate planktonic and biofilm cells (RPMI at pH 4) and to extract the RNA were the same as described above. Then, the complementary DNA (cDNA) was synthesized using the iScript cDNA Synthesis kit (Bio-Rad, Hercules, CA, USA) according to the manufacturer’s instructions. qRT-PCR mixtures were prepared with the cDNA samples, SsoFast EvaGreen Supermix (Bio-Rad, Hercules, CA, USA), and the primers for *ZAP1* and *ACT1*, the latter applied as an internal control. Primers were designed using Primer3 web software (Table S1), and their specificity was confirmed by comparing their sequences to the *Candida* genome database using BLAST (28). The qRT-PCR was performed in a thermocycler (CF X96 Real-Time PCR System; Bio-Rad, Hercules, CA, USA) at 98°C for 2 min, followed by 98°C for 5 s and 57°C for 5 s, during 40 cycles. The relative *ZAP1* expression level was calculated using the ΔCycle threshold (Ct) method (29) and normalized with the *ACT1* internal control gene (Ct_average_ = 25.06 ± 0.28). Each reaction was performed in triplicate.

The same methodology was applied to estimate the transcript levels of *FKS2* and *ERG11* in biofilm cells of *C. glabrata zap1*Δ and *zap1*Δ::*ZAP1* strains, aiming to validate the microarrays results. The primers used for this analysis are presented in Table S1.

### Bioinformatics analyses

The functional description of Zap1 targets (genes and matrix proteins) was obtained with FungiFun 2.2.8 tool (30). A functional enrichment analysis was conducted on FungiFun, using the whole *C. glabrata* genome as background. Additionally, PathoYeastract database (31) was used to search for targets of Zap1 in other fungal species and to retrieve their orthologs in *C. glabrata*. The *Candida* Genome Database (CGD) (32) was used to identify Zap1 targets previously described as required for adhesion, biofilm formation, and virulence (verified and computationally predicted phenotypes) and to categorize the targets according to their molecular activity and cellular localization through the Gene Ontology (GO) Slim Mapper tool. Furthermore, the molecular interactions between Zap1 target proteins were analyzed using STRING 11.0 database (33) and clustered with the Cytoscape tool (34). The minimum required interaction score of STRING analysis was set to 0.4 (medium confidence). The Fungal Secretome Database (FSD) (35) and Fungal Secretome KnowledgeBase (FunSecKB) (36) were used to analyze the predictive secretory nature of Zap1 targets.

### Statistical analyses

The results obtained for *C. glabrata* wild-type, *zap1*Δ and *zap1*Δ::*ZAP1* strains regarding biomass, cell cultivability, biofilm matrix composition, and biofilm thickness were statistically analyzed using the one-way ANOVA and Tukey’s multiple comparisons test, implemented in GraphPad Prism 6 software. The results of *ZAP1* expression in planktonic and biofilm modes (qRT-PCR analysis) were compared with *t* test. Additionally, in the functional enrichment analyses of Zap1 targets performed with FungiFun tool, the statistical Fisher-exact test with Benjamini-Hochberg correction (30, 37), available in the tool, was used. All tests were performed with a confidence level of 95%.

## RESULTS

### Expression of *ZAP1* gene in *C. glabrata*

**Fig 1:**
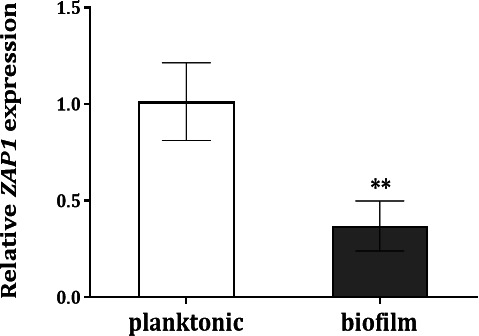
*ZAP1* expression in *Candida glabrata* species. Transcript levels of *ZAP1* gene, estimated by qRT-PCR, in *C. glabrata* wild-type cells grown in planktonic and biofilm modes for 24 h in RPMI at pH 4. The values of the transcript levels were normalized using as internal control the levels of *ACT1* mRNA. The asterisk represents statistical difference in the results (**P-value ≤ 0.01). Error bars represent standard deviation.

The expression level of *ZAP1* gene in *C. glabrata* wild-type cells grown in planktonic and biofilm modes for 24 h, at pH 4, was analyzed using qRT-PCR. The results showed a statistically lower (*P*-value ≤ 0.05) expression of *ZAP1* in biofilm than planktonic cells (Fig. 1).

### Role of Zap1 in *C. glabrata* planktonic growth and biofilm formation

**Fig 2:**
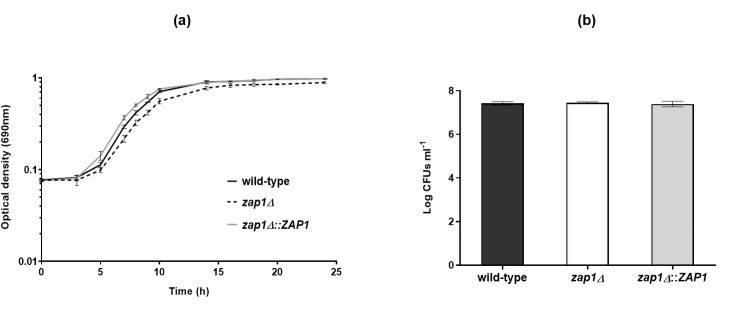
Zap1 role in *Candida glabrata* planktonic growth. (**a**) Growth curves (optical density) and (**b**) cultivable cells (Log CFUs mL^−1^) of *C. glabrata* ATCC 2001 (wild-type), *zap1*Δ, and *zap1*Δ::*ZAP1* cells grown planktonically in RPMI at pH4. Error bars represent standard deviation.

Free-floating cells of the knockout mutant *C. glabrata zap1*Δ, its complemented strain *zap1*Δ::*ZAP1,* and the wild-type strain ATCC 2001, cultured in RPMI at pH 4, showed similar growth rates (Fig. 2a) and similar number of cultivable cells after 24 h of growth (*P*-value > 0.05) (Fig. 2b).

**Fig 3:**
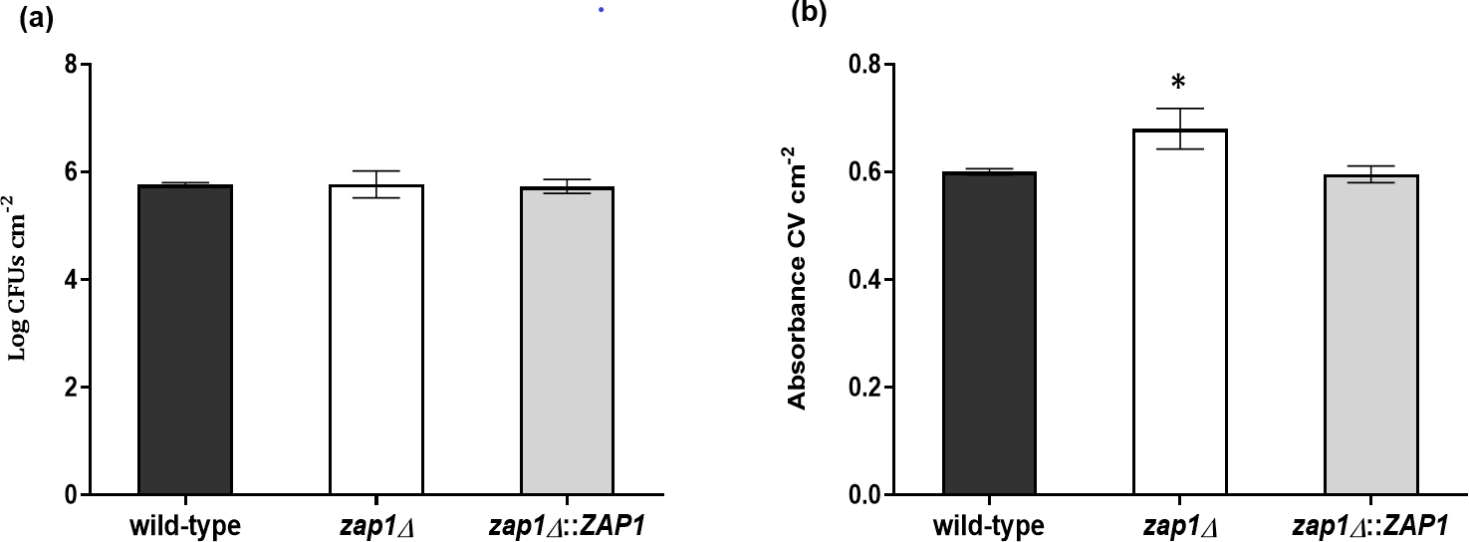
Zap1 role in *Candida glabrata* biofilm formation. (**a**) Cultivable cells [Log (CFUs cm^−2^)] and (**b**) total biomass (Absorbance CV cm^−2^) of *C. glabrata* ATCC 2001 (wild-type), *zap1*Δ and *zap1*Δ::*ZAP1* biofilms developed for 24 h in RPMI at pH4. The asterisk represents the statistical difference between *zap1*Δ and other strains (*P-value ≤ 0.05). Error bars represent standard deviation.

**Fig 4:**
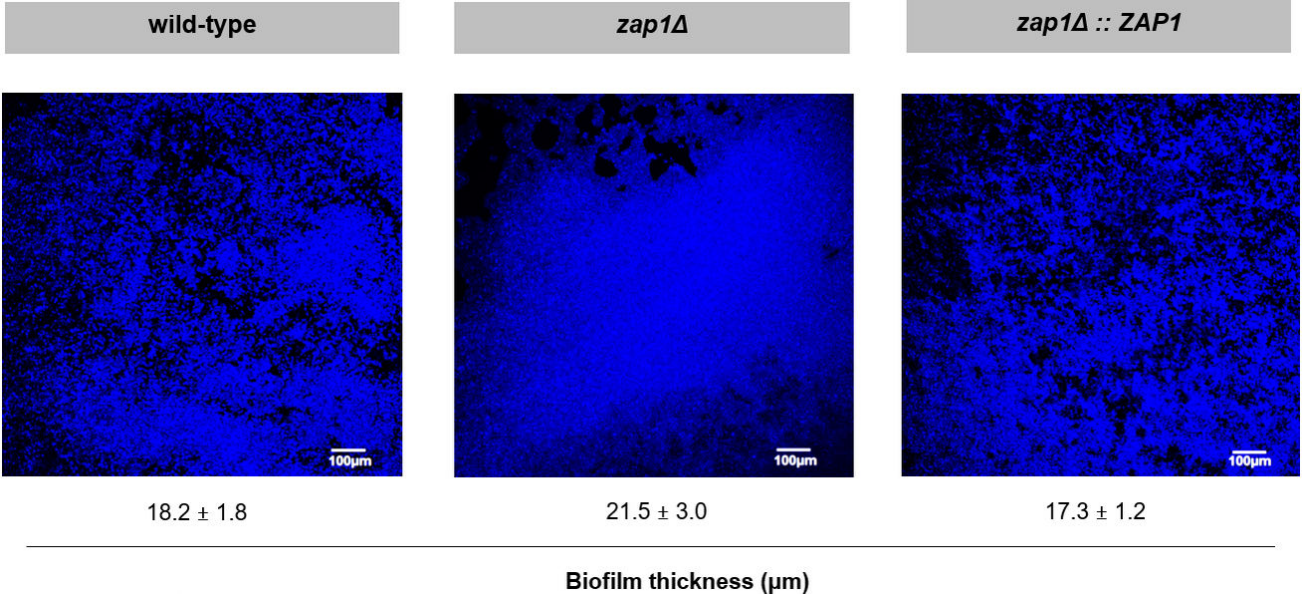
Zap1 role in *Candida glabrata* biofilm structure. Confocal laser microscopy images and thickness (μm) of *C. glabrata* ATCC 2001 (wild-type), *zap1*Δ and *zap1*Δ::*ZAP1* biofilms developed for 24 h in RPMI at pH 4. The original magnification of images was 10×.

Additionally, biofilms of the three strains, developed for 24 h in RPMI at pH 4, presented a similar number of cultivable cells (*P*-value > 0.05) (Fig. 3a); however, differences regarding total biomass (Fig. 3b) and biofilm structure (Fig. 4) were observed. Indeed, z*ap1*Δ biofilm presented a slightly and statistically higher amount of total biomass than those of the other strains (*P*-value ≤ 0.05) (Fig. 3b). Furthermore, the images obtained by CLSM for the wild-type and *zap1*Δ::*ZAP1* biofilms were similar, but z*ap1*Δ biofilm seemed to present a more cohesive structure, with a more uniform “carpet” throughout the image, and a slightly higher thickness than the biofilms of the other strains (Fig. 4).

### Role of Zap1 in *C. glabrata* biofilm matrix production

The matrices of *C. glabrata* wild-type, *zap1*Δ and *zap1*Δ::*ZAP1* biofilms were analyzed in terms of protein, total carbohydrate, and β-glucan amount (Table 1). The amount of protein found in *zap1*Δ biofilm matrix was statistically higher (*P*-value ≤ 0.01) than the amount found in the biofilm matrix of the other strains (Table 1). The total carbohydrate content was also statistically higher (*P*-value ≤ 0.05), presenting more than three times the amount detected in the other two strains. Consistently, the (1,3)-β-d glucan content was also higher (*P*-value ≤ 0.05) in the matrix of *zap1*Δ biofilm although to a lower extent in relation to total carbohydrate (Table 1).

**TABLE 1:** Influence of *ZAP1* deletion on *Candida glabrata* secreted components^*a*^

Sample	Component	*C. glabrata* strain		
		Wild type	zap1Δ	*zap1Δ*::*ZAP1*
Biofilm matrix	Protein (mg/g biofilm)	41.33 ± 4.56	87.16 ± 2.22**	45.44 ± 3.88
	Carbohydrate (mg/g biofilm)	94.08 ± 14.36	368.87 ± 59.03*	106.60 ± 19.62
	(1,3)-β-d-glucan (ng/g biofilm)	45.91 ± 1.16	62.24 ± 5.67*	46.33 ± 0.16
Planktonic supernatant	Protein (µg/mL)	108.85 ± 7.56	120.19 ± 3.40	112.41 ± 5.52
	Carbohydrate (mg/mL)	2.47 ± 0.14	3.16 ± 0.07	2.51 ± 0.24
	(1,3)-β-d-glucan (pg/mL)	104.27 ± 11.67	142.58 ± 13.94	103.71 ± 7.94

^*a*^ Quantification of protein, carbohydrate, and (1,3)-β-d-glucan secreted to the planktonic supernatant and to the biofilm matrix by *C. glabrata* ATCC 2001 (wild-type), *zap1Δ* and *zap1Δ::ZAP1* strains, after 24 h of growth in planktonic or biofilm lifestyles in RPMI at pH 4. The results of the biofilm matrix were normalized with the respective biofilm dry weight, and all the results are presented as means ± standard deviations. Asterisks represent statistical differences between the knockout mutant and other strains (*P-value ≤ 0.05; **P-value ≤ 0.01). Three biological replicates and three technical replicates were included.

The amount of protein and carbohydrate in the supernatant of planktonic cells was also analyzed. The results showed similar amounts of components in the supernatant of the three strains (Table 1).

### Zap1 target genes

Transcriptomic analyses, using dual-channel (control vs test) microarrays, were carried out with cells of *C. glabrata zap1*Δ and *zap1*Δ::*ZAP1* strains, grown in planktonic (control) and biofilm (test) lifestyles, under acidic conditions. These analyses revealed the relative gene expression levels in *zap1*Δ::*ZAP1* and *zap1*Δ strains in the biofilm mode compared to the planktonic mode. The relative expression obtained for the strains was then compared, and genes with higher expression levels in *zap1*Δ::*ZAP1* biofilms than in *zap1*Δ biofilms were selected as Zap1-induced targets, while those in the opposite situation were selected as Zap1-repressed targets. Only genes showing at least a 1.5-fold difference in the relative expression between strains were selected. By performing dual-channel analyses (planktonic vs biofilm) on each strain and then comparing the results, it was ensured that the selected Zap1 targets were biofilm-specific. Biofilm specificity was crucial for this work because the main focus was the molecular changes underlying biofilm matrix regulation.

The results revealed that Zap1 induces the expression of 557 genes and represses 592 genes. Of note, 327 and 364 induced and repressed targets, respectively, displayed a strong regulation (above 2-fold change). A set of Zap1 target genes with the strongest regulation is presented in Table 2. The full list of targets, their expression level in each strain, and ratio between strains is presented in Table S2. The results of the microarrays experiments were validated through qRT-PCR for two Zap1 targets, *FKS2* (involved in glucan metabolism) and *ERG11* (involved in antifungal resistance). The results confirmed their induction by Zap1 (Fig. S3).

**TABLE 2:** Target genes of Zap1 found in *C. glabrata* acidic biofilms^*a*^

Zap1-induced genes		Zap1-repressed genes	
Gene name	Expression level	Gene name	Expression level
*DUR31*	14.608	*CAGL0L00429g*	0.035
CAGL0M03377g*	9.942	*EPA20*	0.053
*CAGL0E05984g*	8.778	*SUT1*	0.059
*CAGL0L03982g*	8.532	*CAGL0J00715g*	0.061
HXK2*	8.137	*CAGL0F04521g*	0.076
*CAGL0L00649g*	7.947	*CAGL0E01353g*	0.095
*CAGL0J05852g*	7.739	*PHM8*	0.103
*PEX21*	7.295	*CAGL0J10296g*	0.117
*CAGL0G08668g*	7.243	*CAGL0F04499g*	0.118
*MET6*	6.746	*CAGL0F05137g*	0.127
*UTR2*	6.343	*CAGL0M01870g*	0.128
CAGL0K00825g*	6.164	*HAP4*	0.128
*CAGL0J08569g*	5.956	*FES1*	0.130
CAGL0D00198g*	5.941	*CAGL0K07315g*	0.133
*CAGL0D05742g*	5.907	*AQR1*	0.133
*CAGL0I02178g*	5.818	*CAGL0D05632g*	0.134
*CAGL0B04543g*	5.781	*CAGL0J04554g*	0.142
*CAGL0L11440g*	5.467	*RSB1*	0.143
*CAGL0B02651g*	5.307	*CAGL0K07205g*	0.147
*GSH2*	5.301	*TPN1*	0.149

^*a*^ The 20 genes found to have the highest (Zap1-induced) and lowest (Zap1-repressed) expression ratio between *zap1*Δ::*ZAP1* biofilm and *zap1*Δ biofilm are here listed. Zap1-target genes coding Zap1-target proteins are marked with *. The full list of Zap1 targets, expression level in each strain, and ratio are presented in Table S2.

### Zap1 target matrix proteins

**Fig 5:**
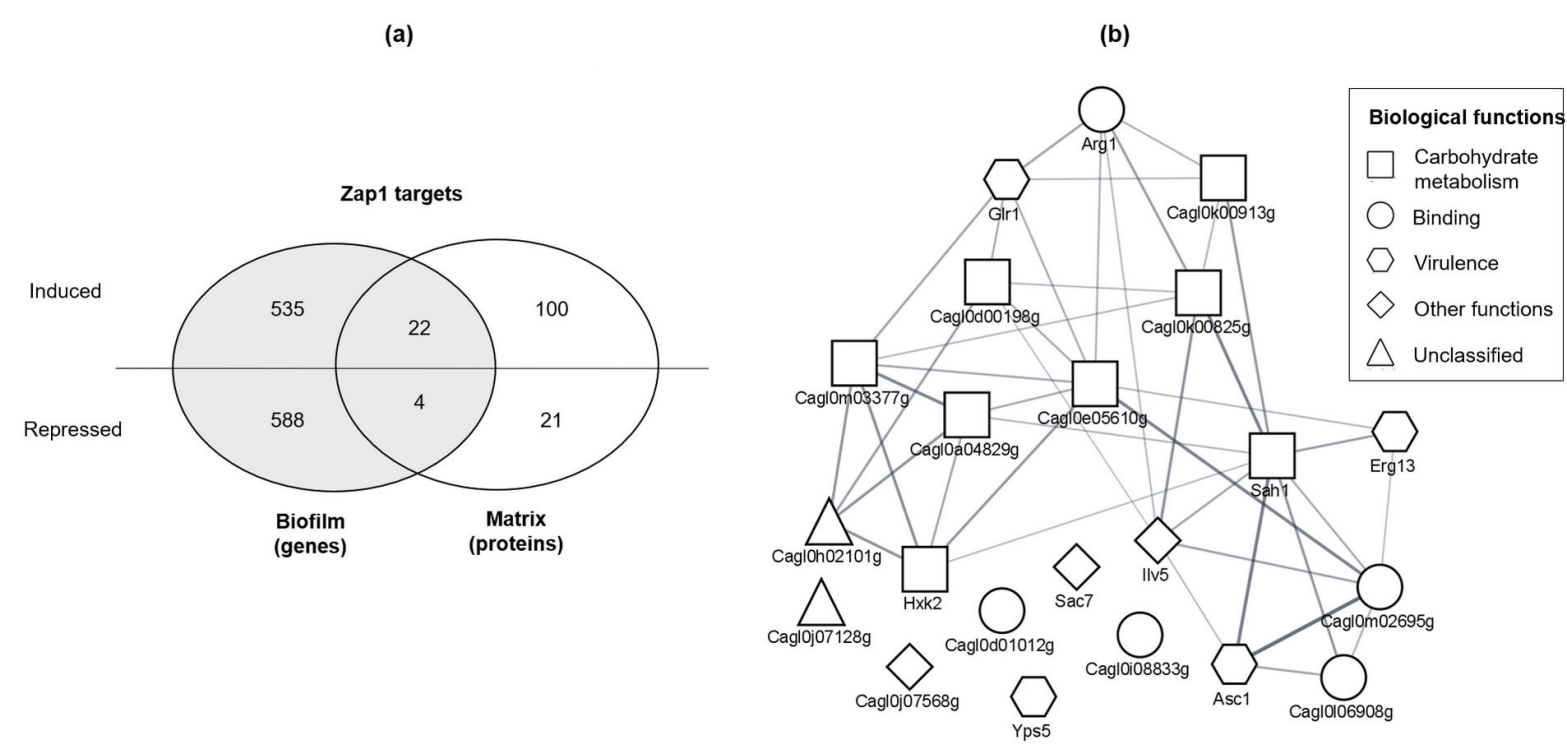
Overlap between Zap1 target biofilm genes and Zap1 target matrix proteins. (**a**) Venn diagram showing the overlap between the proteins coded by Zap1 target biofilm genes and Zap1 target matrix proteins. (**b**) Zap1-induced matrix proteins overlapping with proteins coded by zap1-induced genes are herein distributed according to their molecular interactions annotated in STRING and clustered with the Cytoscape tool. Geometrical forms represent proteins and the respective biological function annotated in FungiFun database.

The proteins in the matrix of *zap1*Δ::*ZAP1* and *zap1*Δ biofilms were identified, by LC-MS/MS. Then, proteins exclusively found in the matrix of *zap1*Δ::*ZAP1* biofilm were selected as Zap1-induced and those found only in *zap1*Δ biofilm matrix as Zap1-repressed. The results showed that Zap1 induced and repressed the secretion of 122 and 25 proteins to the biofilm matrix, respectively (Fig. 5a). The full list of Zap1 target matrix proteins is provided in Table S2, and a subset of Zap1-induced proteins is presented in Fig. 5b.

### Bioinformatics analysis of Zap1 targets

#### Target biofilm genes versus target matrix proteins

The sets of Zap1-induced and -repressed genes, identified through microarrays, were compared with the respective sets of Zap1 target matrix proteins (i.e., their encoding genes), identified by LC-MS/MS, using a Venn diagram. This analysis revealed an overlap of 22 and 4 induced and repressed targets, respectively (Fig. 5a).

#### Molecular interaction and activity

The molecular interactions among Zap1 targets were predicted using STRING database (33). This analysis revealed statistical enrichment of protein-protein interactions among all sets of targets analyzed, except for matrix proteins repressed by Zap1 (Table 3). According to STRING, this statistical enrichment means that the expected number of interactions for a random set of proteins of similar size is lower than that obtained (Table 3) (33). Additionally, the subset of 22 Zap1-induced matrix proteins overlapping with Zap1-induced genes (Fig. 5a) was also analyzed in STRING and clustered in Cytoscape (34), and the biological functions were obtained in FungiFun database (30) (Fig. 5b). This subset of proteins also presented a statistical enrichment of protein-protein interactions (*P*-value < 0.001).

**TABLE 3:** Molecular interactions between Zap1 targets^*a*^

STRING network results	Biofilm targets		Matrix targets	
	Induced	Repressed	Induced	Repressed
Total regulated proteins	557	592	122	25
Number of obtained interactions	7,701	3,924	933	4
Number of expected interactions	6,338	2,838	590	5
Average protein degree	27.7	13.3	15.3	0.32
Avg. local clustering coefficient	0.315	0.298	0.47	0.32
PPI enrichment *P*-value	<1.0e−16	< 1.0e-16	< 1.0e-16	0.756

^*a*^ Results of STRING analysis of the molecular interactions among the proteins coded by Zap1 target biofilm genes (biofilm targets) and among Zap1 target matrix proteins (matrix targets). The minimum confidence interaction score was set to 0.4 (medium). PPI means protein–protein interaction.

A gene ontology (GO) analysis was carried out to categorize Zap1 targets according to their molecular activity annotated in CGD (manually curated and predicted computationally by orthology) (32). The results revealed that Zap1-induced and -repressed genes are involved in 20 and 21 molecular activities, respectively, and Zap1-induced and -repressed matrix proteins in 19 and 11 activities, respectively (Table S3). Transferase was the most enriched activity among all sets of targets analyzed, annotated to ~21% and 17% of Zap1-induced genes and matrix proteins, respectively, (Table 4) followed by hydrolase activity (Table S3).

**TABLE 4:** Bioinformatics analyses of Zap1 targets^*a*^

	Biofilm targets		Matrix targets	
	Induced *N* (%)	Repressed *N* (%)	Induced *N* (%)	Repressed *N* (%)
**Total**	**557**	**592**	**122**	**25**
Predicted transferase activity (24) Table S3	115 (20.6)	69 (11.7)	21 (17.2)	3 (12.0)
Involved in carbohydrate metabolism (24) Table S4	87 (15.6)	28 (4.7)	20 (16.4)	3 (12.0)
Reported Zap1 gene targets in *C. albicans* (38) Table S5	27 (4.8)	31 (5.2)	9 (7.4)	3 (12.0)
Reported Zap1 gene targets in *S. cerevisiae* (38) Table S5	42 (7.5)	35 (5.9)	13 (10.7)	2 (8.0)
Reported Zap1 targets in *C. albicans* biofilm (15) Table S5	24 (4.3)	23 (3.9)	9 (7.4)	3 (12.0)
Predicted cytoplasm localization (24) Table S6	290 (52.1)	213 (35.9)	64 (52.5)	10 (40.0)
Secretory nature (26) Table S5	231 (41.5)	254 (42.9)	59 (48.4)	16 (64.0)

^*a*^ Results of the bioinformatics analyses performed with genes (biofilm targets) and matrix proteins (matrix targets) regulated by Zap1 in *C. glabrata* biofilms, including gene ontology (molecular activity and cellular localization), biological function, previous report as Zap1 targets in other fungal species (by orthology), and predicted secretory nature. The results that gave rise to this table are fully detailed in Tables S3 to S6.

#### Functional distribution

**Fig 6:**
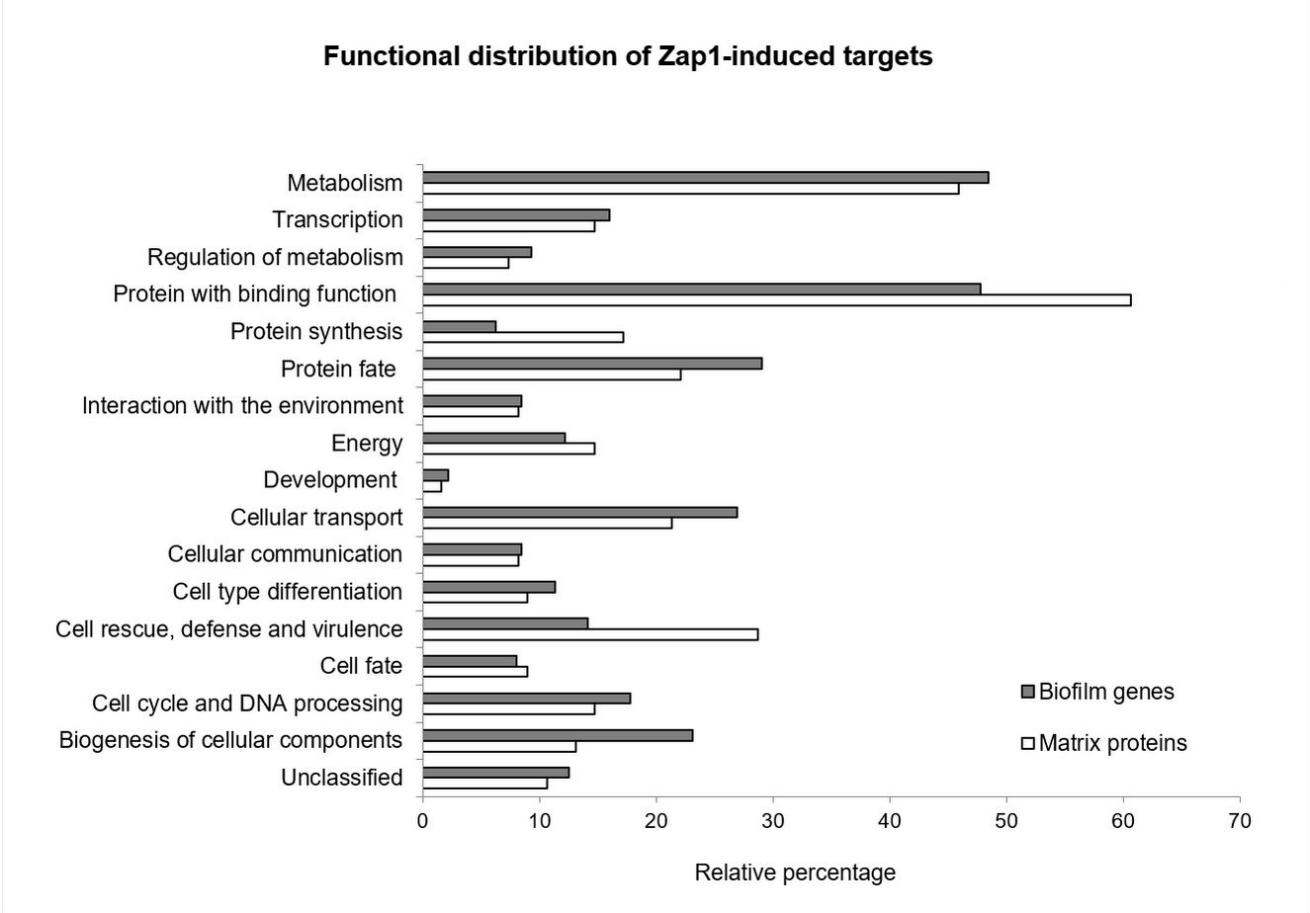
Functional distribution of *C. glabrata Zap1*-induced targets. Biofilm genes and matrix proteins found to be induced by Zap1 in *C. glabrata* biofilms were clustered according to their predicted biological function according to FungiFun database. The relative percentage shown corresponds to the number of targets included in each functional class compared to the total number of Zap1-induced biofilm genes and Zap1-induced matrix proteins. The results that gave rise to this figure are presented in Table S4.

The functional distribution of Zap1 targets found in this study was analyzed using FungiFun tool (30). This analysis revealed an enrichment of functions related to “metabolism” and “protein with binding function” in the data sets of induced targets (genes and matrix proteins), followed by the “cellular transport” and “cell rescue, defense and virulence” functions (Fig. 6). Around ~10% of induced targets had an unclassified function. The functional distribution of Zap1-repressed targets was, in general, similar to that of induced targets but with lower relative percentage associated to each functional class (Fig. S4). Furthermore, 25% and 36% of Zap1-repressed genes and matrix proteins, respectively, have an unclassified function (Fig. S4).

The analysis of functional subclasses revealed a statistical enrichment (relative to the whole *C. glabrata* genome) of 23 and 17 subfunctions in Zap1-induced and -repressed genes, respectively, and of 10 and 1 subfunctions in Zap1-induced and -repressed matrix proteins, respectively (Table S4). The subfunctions with the most significant statistical result in the set of induced genes belong to “metabolism” and “energy” main functions, including “carbohydrate metabolism” (~16% of targets; Table 4), “amino acid metabolism,” “glycolysis,” and “fermentation” (Table S4). Regarding Zap1-induced matrix proteins, the most enriched subclasses included “translation” and “stress response.” Among Zap1-repressed targets subclasses, such as “sugar binding,” “sugar transport,” “regulation of carbohydrate metabolism,” and “adhesion” were found to be enriched (Table S4),

#### Overlap with Zap1 targets of other fungal species

All Zap1 target genes annotated to *C. albicans* and *Saccharomyces cerevisiae* were retrieved in PathoYeastract (20) and compared with Zap1 targets (genes and matrix proteins) found in this study (by ortholog alignment). This analysis revealed an overlap with the targets reported in *C. albicans* and *S. cerevisiae* ranging between 5% and 12% (Table 4). The list of *C. glabrata* Zap1 targets overlapping with targets reported in *C. albicans* and *S. cerevisiae* species are described in Table S5.

#### Cellular localization and secretory nature

A GO analysis was carried out to categorize Zap1 targets according to their predicted cellular localization annotated in CGD (manually curated and predicted) (32). The results revealed that Zap1-induced and -repressed genes are annotated to 23 and 22 different cellular locations, respectively, and Zap1-induced and -repressed matrix proteins to 21 and 17 locations, respectively (Table S6). The most enriched cellular localization was the cytoplasm, annotated to around half of all sets of targets (Table 4), followed by the membrane (Table S6).

The predictive secretory nature of Zap1 targets, including the presence of an hydrophobic signal sequence at the N-terminus and/or a C-terminal sequence for Glycosylphosphatidylinisotol (GPI) modification (39), was analyzed using FSD (35) and FunSecKB (36) databases. These analyses showed that ~42% and 48% of Zap1-induced genes and matrix proteins, respectively, have a predictive secretory nature according to at least one of the databases (Table 4; Table S5).

#### Putative phenotype analysis

**Fig 7:**
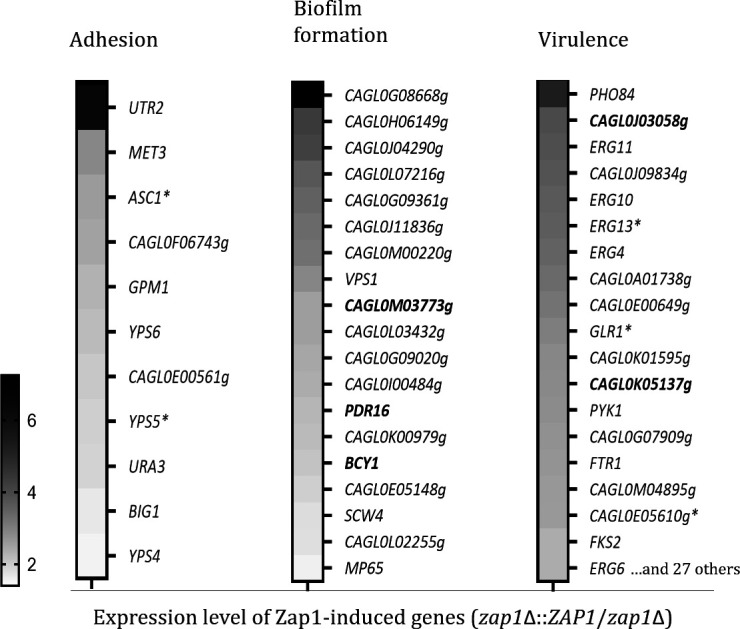
Zap1-induced targets with potential roles in adhesion, biofilm formation, and virulence. Zap1-induced genes annotated to the phenotypes “adhesion,” “biofilm formation,” and “virulence” in CGD for *C. glabrata* species (highlighted in bold) and other *Candida* species (by orthology) (Table S5), and respective expression level ratio, as estimated by microarrays (*zap1*Δ::*ZAP1*/*zap1*Δ) (Table S2). Target genes encoding target matrix proteins are marked with the asterisk.

The predicted phenotypes annotated to Zap1 targets were searched in CGD (32), using the key terms “adhesion,” “biofilm,” and “virulence” (Table S5). This analysis revealed that 11, 19, and 46 Zap1-induced genes are annotated as required for adhesion, biofilm, and virulence, respectively, in *C. glabrata* or other *Candida* species (by orthology). These target genes are presented in Fig. 7 with the respective expression level ratio (*zap1*Δ::*ZAP1*/*zap1*Δ). The targets in which the associated phenotype was reported in *C. glabrata* species are highlighted in bold (the remaining were associated with the phenotypes in other species, by orthology) and those encoding matrix proteins found to be Zap1-induced are marked with the asterisk (*). Targets associated with more than one of the phenotypes were presented only in one group. All Zap1 target genes annotated to the phenotypes, and the respective *Candid*a species where the phenotype was reported, are presented in Table S5.

## DISCUSSION

We have previously demonstrated that *C. glabrata* biofilm formation is enhanced by vaginal acidic conditions and that the composition of the biofilm matrix, including the proteome, is highly dependent on environmental pH (8, 12). With the aim of deepening knowledge about the regulation of the matrix of *C. glabrata* biofilms developed under acidic conditions, we studied the transcription factor Zap1, an ortholog of a *C. albicans* biofilm matrix regulator. To our knowledge, this is the first study to reveal the role of Zap1 in the *C. glabrata* biofilm matrix and the Zap1 targets in this species, including genes and matrix proteins.

According to our data, the role of Zap1 as a negative regulator of biofilm matrix production reported in *C. albicans* (15) is conserved in the *C. glabrata* species. In fact, the deletion of *ZAP1* gene from the *C. glabrata* genome did not affect the growth rate or the cultivability of planktonic cells (Fig. 2) or the biofilm formation ability in terms of cell cultivability (Fig. 3a). However, a slightly greater amount of biofilm biomass (Fig. 3b), along with greater biofilm cohesion and thickness (Fig. 4), was observed in *zap1*Δ strain, suggesting a potential greater accumulation of biofilm matrix components. Indeed, a significantly higher amount of total carbohydrate, (1,3)-β-d-glucan, and protein was found in the biofilm matrix of the knockout *zap1*Δ mutant, but the same was not observed for planktonic cells (Table 1). Importantly, the expression level of the *ZAP1* gene was lower in biofilm than in planktonic cells under acidic conditions (Fig. 1), which may occur to minimize its negative impact on matrix production, as previously suggested for *C. albicans* (15).

The similar function of Zap1 in both species made us question whether the molecular mechanisms underlying the regulation of the biofilm matrix are also conserved. Zap1 was found to be a relevant regulator of the biofilm transcriptome (557 up- and 592 downregulated genes), as reported for *C. albicans* (15). However, the Zap1 targets found in this study showed a low overlap with those reported in *C. albicans* biofilms (by orthology) (Table 4). In fact, low overlap was found with all Zap1 targets reported for *C. albicans* and *S. cerevisiae* (5%-12%; Table 4). Several factors may have contributed to this low overlap including (i) in this study, only Zap1 targets specifically regulated in the biofilm mode were selected (the planktonic mode was used as a control in the dual-channel microarrays analysis); (ii) our experiments were carried out under specific acidic conditions; (iii) the lack of filamentation capacity by *C. glabrata* species, which is regulated by Zap1 in *C. albicans;* (iv) the recruitment of different molecular machinery for similar processes. Indeed, despite the similar role of Zap1 as a negative regulator of carbohydrate accumulation in the matrix of *C. glabrata* and *C. albicans* biofilms, the machinery recruited for this process may differ between species. For instance, Zap1 has been found to repress several genes involved in the “carbohydrate transport” function, in both species, which may contribute to its role as negative regulator of matrix carbohydrate accumulation (Table S4) (15); however, no overlap in the targets annotated for this function was observed. Additionally, *ZRT* family orthologs, which is known to be induced by Zap1 in *C. albicans* (15), were not induced by Zap1 in this study. However, other genes involved in zinc homeostasis such as *CAGL0E02519g* and *CAGL0D00176g* were induced by Zap1 (Table S5). Nevertheless, the experimental setting of this study, particularly the acidic pH, may have had a significant impact on the results, as Zap1 targets reported for other species were identified in neutral or near neutral pH conditions. Furthermore, we recently demonstrated that environmental pH has a significant impact on the matrix proteome of *C. glabrata* biofilms (12). Importantly, 30% matrix proteins induced by Zap1 correspond to proteins previously reported to be secreted under acidic conditions, but not at neutral pH (12). This result suggests that Zap1 may also be involved in the response to the acidic environment.

The bioinformatics analyses performed here pointed to a complex role of Zap1 in the matrix regulation, acting both as negative and positive regulator. Zap1 was found to repress genes/matrix proteins involved in “regulation of carbohydrate metabolism,” “sugar binding,” and “sugar transport,” which may contribute to impair the delivery of carbohydrates to the matrix and, thus, to its role as negative regulator of matrix accumulation. Various cell wall proteins with role in adhesion, including those of Epa family (Epa1, Epa3, Epa6, and Epa22), were also found among Zap1-repressed targets. Cell wall proteins have been suggested to intermediate the covalent linkages between exopolysaccharides similar to what occurs in the cell wall (40), and their repression may contribute to a looser matrix. However, a high enrichment of the “carbohydrate metabolism” function was found in the set of Zap1-induced genes (Table 4). Indeed, various targets were found to have a potential involvement in glucan (e.g., Scw4, Fks2, Gdb1 Cagl0M03377g) and mannan (e.g., Mnn2, Bmt6, Sec53, Vig9) metabolism, mostly hydrolases and transferases. This result suggests a potential role of Zap1 in the delivery and organization of carbohydrates in the biofilm matrix (12, 14). Additionally, Zap1 was required for the secretion of 122 proteins into the matrix. As such, despite being a negative regulator of matrix amount, Zap1 seems to be required for the delivery and organization of some matrix components, pointing to a complex regulation.

According to the bioinformatics analyses, Zap1 may also be involved in relevant roles such as energy generation, adhesion, virulence, antifungal resistance, and host immunity evasion (Fig. 7). Several yapsins were found to be induced by Zap1 (Yps3, Yps4, Yps5, Yps6, Yps7, and Yps11), which are orthologs of *C. albicans* secreted aspartyl proteinases, known to be required for adhesion (41). Yapsins have also been reported to be essential for virulence and to have a role in pH and vacuole homeostasis, cell wall integrity, energy production, survival in macrophages, and survival in the presence of weak acids (including lactic acid) (42, 43). The *ERG* family found to be induced by Zap1 (Erg1, Erg3, Erg4, Erg6, Erg8, Erg10, Erg11, Erg13, Erg20) has been reported as required for virulence and essential for antifungal drug resistance, cell wall integrity, cell viability, and growth (44–47). Zap1 also induced various genes involved in energy-related functions, such as glycolysis and fermentation, pointing to a role in the assimilation of carbohydrates for generating energy and producing biomolecules, thus contributing for biofilm cells survival and growth (48). Of note, although Zap1 targets are associated with several biological functions (Fig. 6), a predicted close molecular interaction, either direct or indirect, was found among them (Table 3), indicating that they are biologically connected (33). As such, although the matrix composition of *C. glabrata* biofilms is multifaceted in many aspects, most Zap1-regulated proteins identified in the matrix are putatively functionally connected with one or more molecules within the matrix proteome (Fig. 5b).

Interestingly, Zap1 was found to induce moonlighting enzymes involved in glycolysis (Pgk1 and Tkl1) or other central metabolic pathways (Gnd1, Ilv5, and Pgi1) and proteins with a role in intracellular stress response (Cta1, Glr1, and Ahp1) (49). Moonlighting proteins are primarily intracellular but also display relevant extracellular functions when secreted by unconventional pathways, such as vesicles (12, 50). Indeed, more than 50% of Zap1 targets do not have a predicted secretory nature (Table 4), thus being secreted by unconventional pathways. Moonlighting proteins play important extracellular functions, including adhesion (51, 52), immune system evasion (53, 54), response to the oxidative stress (55, 56), and stabilization of biofilms (38, 57). Despite the potential of moonlighting proteins as targets for the development of novel therapeutics, most of these proteins, such as those involved in glycolysis, possess catalytic mechanisms conserved in human hosts. Thus, blocking their secretion has been suggested the most suitable strategy (58, 59). Our results indicate that Zap1 may be an interesting target for the development of novel therapies and to control *C. glabrata* acidic biofilms.

**Fig 8:**
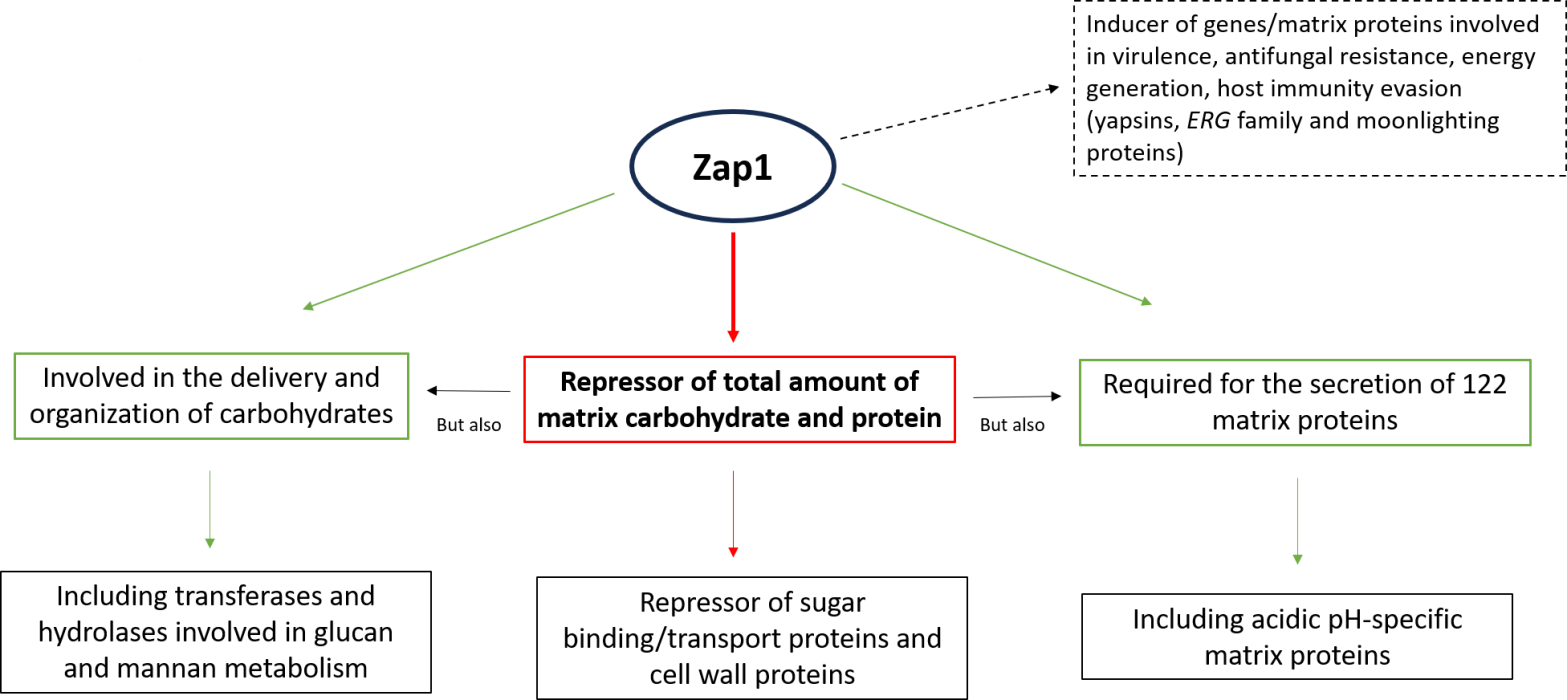
Schematic representation of biofilm matrix regulation by *Candida glabrata* Zap1 under acidic conditions. This study demonstrated that Zap1 acts as a negative regulator of the total amount of matrix components (Table 1), supported by the repression of proteins involved in sugar binding/transport and cell wall proteins involved in the carbohydrates’ linkages. However, Zap1 is essential for the secretion of several matrix proteins (Fig. 5), including those specific for acidic pH, and plays a potential role in the delivery and organization of matrix carbohydrates, as indicated by the induction of transferases and hydrolases involved in glucan and mannan metabolism. In addition, this study reveals that Zap1 triggers the expression of genes and secretion of matrix proteins with diverse functions, including virulence, energy production, antifungal resistance, and evasion of host immunity. The high degree of molecular interaction among Zap1-induced targets (Table 3) suggests possible functional synergy or cooperation that enhances the efficiency of their biological processes, also implying the formation of protein networks.

The study’s most significant findings were consolidated in Fig. 8, which depicts a schematic representation of how *C. glabrata* Zap1 potentially regulates the biofilm matrix under acidic conditions.

### Conclusions

This study revealed that Zap1 is a complex matrix regulator of *C. glabrata* acidic biofilms, with a relevant impact on the transcriptome and matrix proteome. Zap1 was found to be a negative regulator of the amount of protein and carbohydrate in the biofilm matrix. However, it was also found to be required for the secretion of various matrix proteins and seems to contribute to the delivery and organization of matrix carbohydrates. Furthermore, Zap1 may contribute to the generation of energy by the biofilm cells, virulence, adhesion, resistance to antifungals, host immunity evasion, and response to the acidic environment. This study suggests that Zap1 may be an interesting target for novel therapeutics, and thus, further studies, including *in vivo*, would be essential to ascertain the relevance of this transcription factor. Although many questions remain, the high-throughput and bioinformatics analyses described here represent a major step toward a better understanding of *C. glabrata* biofilm matrix regulation and will serve as an excellent framework for future studies.

## Data Availability

The microarrays data have been deposited in GEO (identifier GSE226915) (60). The mass spectrometry proteomics data have been deposited to the ProteomeXchange Consortium via the PRIDE (61) partner repository, with the identifier PXD044153.

## SUPPLEMENTAL MATERIAL

Supplemental figures spectrum.01201-24-s0001.pdf Fig. S1 to S4.

Table S1 spectrum.01201-24-s0002.pdf Primers used in this study.

Table S2 spectrum.01201-24-s0003.xlsx Lists of Zap1 targets found in *Candida glabrata* acidic biofilms.

Table S3 spectrum.01201-24-s0004.xlsx Molecular activity of *Candida glabrata* Zap1 targets.

Table S4 spectrum.01201-24-s0005.xlsx Functional distribution of *C. glabrata* Zap1 targets.

Table S5 spectrum.01201-24-s0006.xlsx Bioinformatics analyses of Zap1 targets.

Table S6 spectrum.01201-24-s0007.xlsx Cellular localization of *Candida glabrata* Zap1 targets.

ASM does not own the copyrights to Supplemental Material that may be linked to, or accessed through, an article. The authors have granted ASM a non-exclusive, world-wide license to publish the Supplemental Material files. Please contact the corresponding author directly for reuse.
