# Supplementary material for: Biofilm matrix regulation by Candida glabrata Zap1 under acidic conditions: transcriptomic and proteomic analyses
Source: Microbiol Spectr. 2024 Nov 4;12(12):e01201-24. doi: 10.1128/spectrum.01201-24 (PMC11619577; doi:10.1128/spectrum.01201-24)
Supplement: Supplemental figures — Fig. S1 to S4. [file spectrum.01201-24-s0001.pdf]

(a)

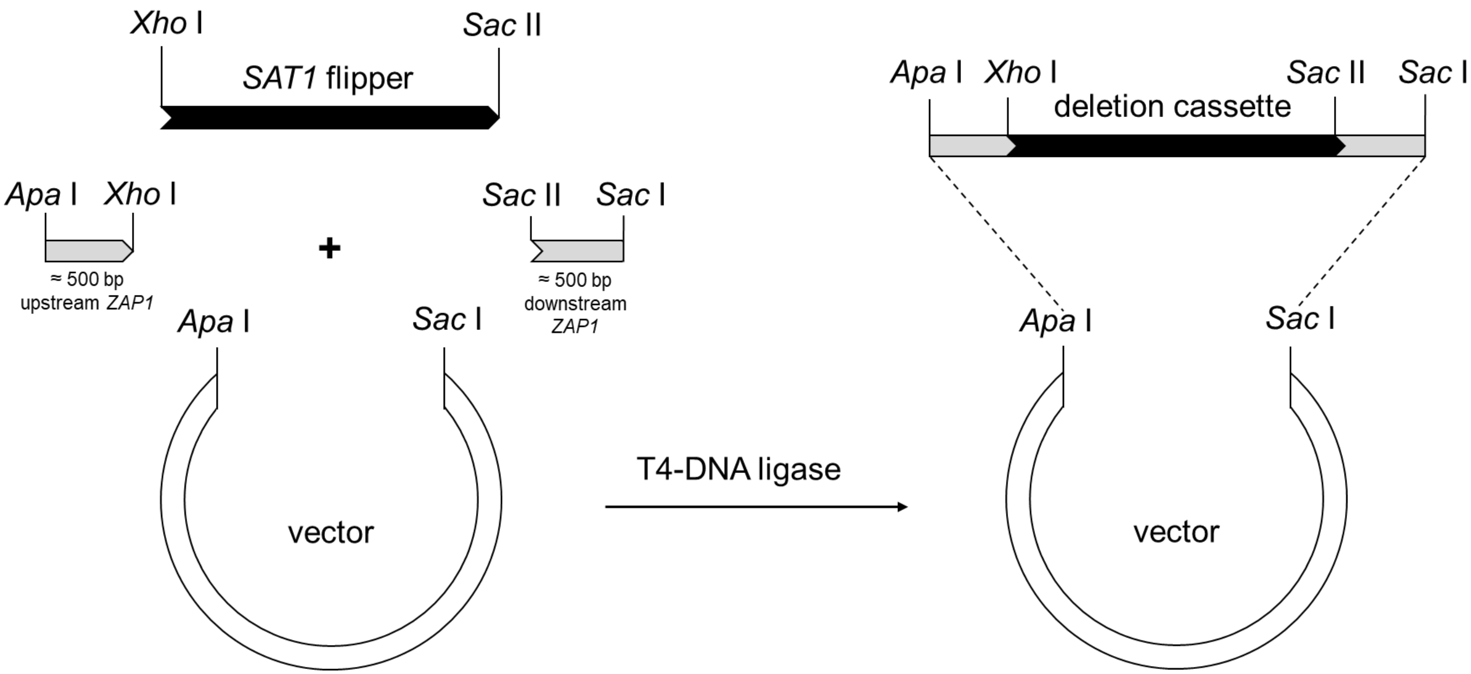

Figure S1. Schematic representation of *C. glabrata zap1*Δ mutant construction. (a) Quadruple ligation performed for cloning the upstream and downstream sequences of *ZAP1* into a plasmid containing the *SAT1* flipper cassette, generating a plasmid construct containing the deletion cassette.

(b)

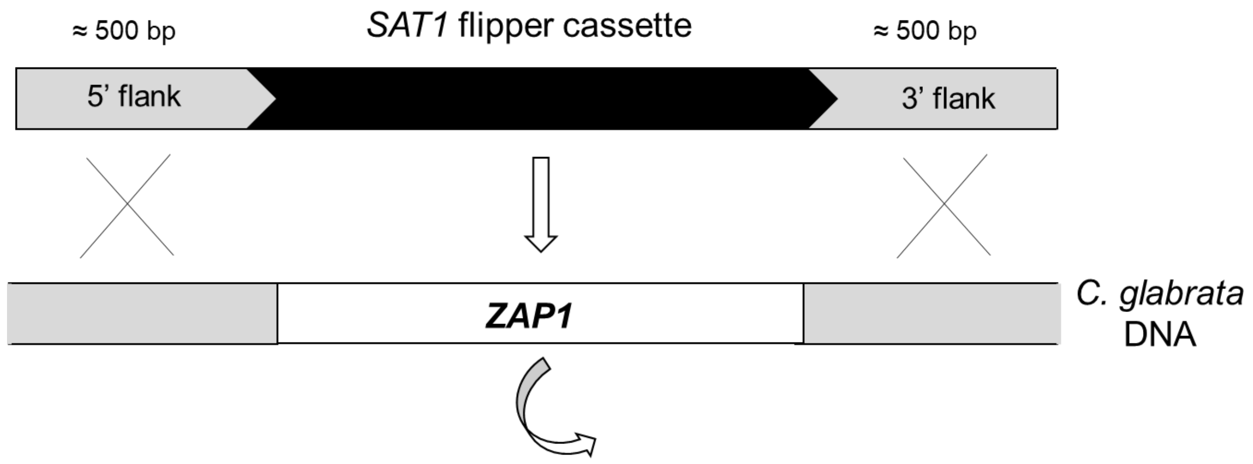

Figure S1 (Continuation). Schematic representation of *C. glabrata zap1*Δ mutant construction. (b) Homologous recombination between the flanking sequences of the deletion cassette and the sequences flanking the *ZAP1* gene in *C. glabrata* genome, leading to the replacement of *ZAP1* gene by the *SAT1* flipper cassette**.**

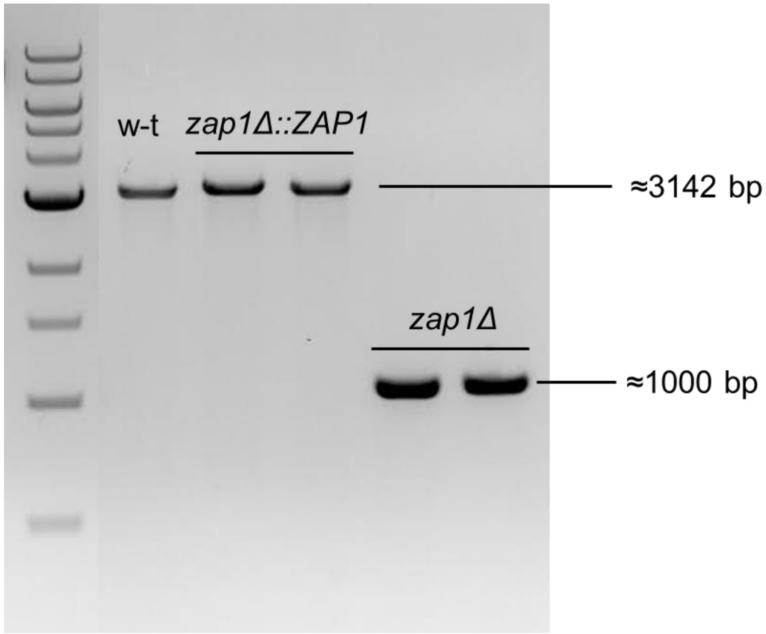

Figure S2. Confirmation of mutant strains construction. Fragments' size obtained after the amplification of *ZAP1* gene (2142 bp) plus its flanking sequences (≈1000 bp), performed by PCR in *C. glabrata* ATCC 2001 (w-t), *zap1Δ* mutant and its complemented strain *zap1Δ::ZAP1*.

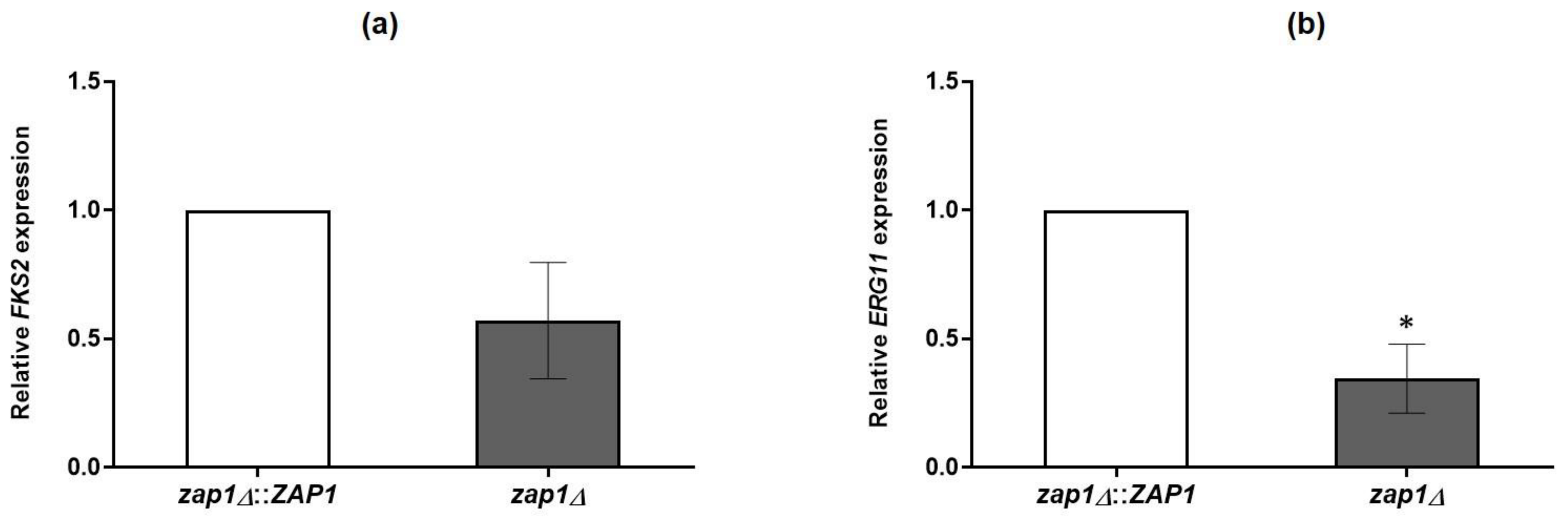

Figure S3. Microarrays validation by qRT-PCR. Transcript levels of **(a)** *FKS2* and **(b)** *ERG11*, estimated by qRT-PCR, in *C. glabrata zap1Δ* and *zap1Δ::ZAP1* biofilms developed for 24 h in RPMI at pH4. The values of the transcript levels were normalized using as internal control the levels of *ACT1* mRNA. Asterisk represent statistical difference in the result of the mutant strain in relation to the complemented strain (* P-value ≤0.05).

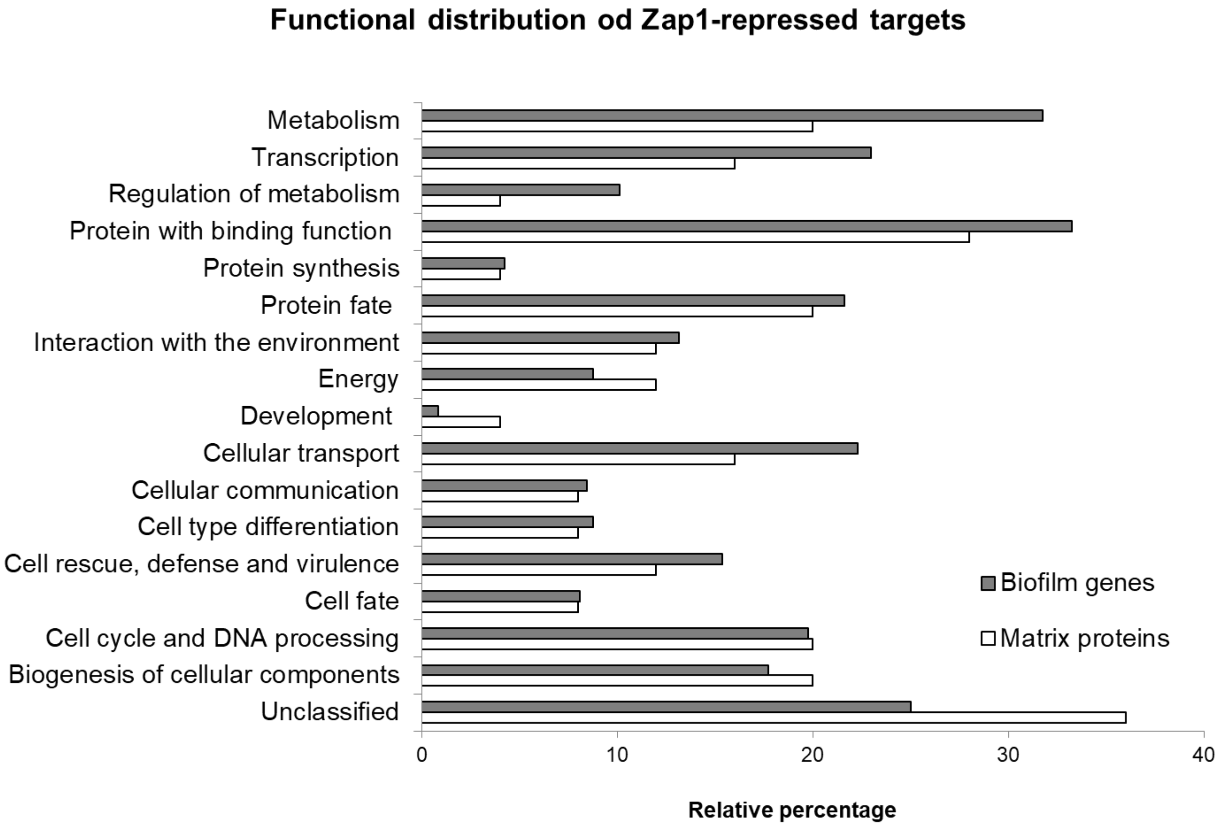

Figure S4. Functional distribution of *C. glabrata Zap1*-repressed targets. Biofilm genes and matrix proteins found to be repressed by Zap1 in *C. glabrata* biofilms, were clustered according to their predicted biological function, according to FungiFun database. The relative percentage shown correspond to the number of targets included in each functional class compared to the total number of Zap1-repressed biofilm genes and Zap1-repressed matrix proteins.
