# Supplementary material for: Biofilm matrix regulation by Candida glabrata Zap1 under acidic conditions: transcriptomic and proteomic analyses
Source: Microbiol Spectr. 2024 Nov 4;12(12):e01201-24. doi: 10.1128/spectrum.01201-24 (PMC11619577; doi:10.1128/spectrum.01201-24)
Supplement: Table S1 — Primers used in this study. [file spectrum.01201-24-s0002.pdf]

Table S1. Primers used in this study

Primer	Sequence (5´→ 3´)	Description
ACT1_Fw	GACGCTCAGTGCACACAACT	To amplify *ACT1* gene*
ACT1_Rv	GCAAAACCGGCTTTACACAT	
FKS2_Fw	GGGTCACTGTGAAATGTT	To amplify *FKS2* gene*
FKS2_Rv	GTAGACGGGTTCGGATT	
ERG11_Fw	CTCCATACTTGCCATTCGGT	To amplify *ERG11* gene*
ERG11_Rv	CTTCAGTTGGGTAACGCCAT	
ZAP1_Fw	TTGATGTGTTTGGCCATGGG	To amplify *ZAP1* gene*
ZAP1_Rv	ACAACAACCGCATCCACTTC	
ZAP1_before_Fw	AGCTGGGCCCGCAAGAGGGACAAATCGTCGG	To amplify the upstream flanking sequence of *ZAP1* gene
ZAP1_before_Rv	AGCTCTCGAGCGTTATCCTCTTGTACCCAAGCC	
ZAP1_after_Fw	AGCTCCGCGGCGGACACCTACTGCACCTCTG	To amplify the downstream flanking sequence of *ZAP1* gene
ZAP1_after_Rev	AGCTGAGCTCGCTCTACTCTCGCATTGATCTGC	
SAT1_Fw	GGAGCGATAAGCGTGCTTCTGC	To amplify the deletion cassette
SAT1_Rv	CCACCTGCTCAGGGATCACC	
ZAP1_P1_ Fw	CTGGAAAGTAAGAACCGCTGGC	To confirm the construction of the deletion cassette
ZAP1_P1_ Rv	GCATCAACCGGTGCTCCTGG	
ZAP1_P2_ Fw	AGGAGAAGCGATGAACCCAATTCGCCCTATAGT GAGTCG	
ZAP1_P2_ Rv	TATGCCCTTGCTTCTGGCGGCCCGGTACCCAG CTTTTG	
ZAP1_Compl_Fw	AGCTCCGCGGATGGTGGAAGGAGTAGTGCACG	To amplify the flanking sequence of *ZAP1* plus the gene
ZAP1_Compl_Rv	AGCTCTCGAGCTAGGTGGCAATCTGCTGGTCC	
SAT1_Compl_Fw	ACATGGTTTATGTGATCGAGGAAGTTCCTATACT TTCTAGAG	To amplify the complemented deletion cassette
SAT1_Compl_Rv	GAAATGTGTGTCTCTCTGGCGGTGGCGGCCGC TCTA	
ZAP1_P3_ Fw	GCAAGAGGGACAAATCGTCGG	To confirm the construction of the complemented deletion cassette
ZAP1_P3_ Rv	GCTCTACTCTCGCATTGATCTGC	
ZAP1_P4_ Fw	GCTTTTGGGCTCATCGCTTCGC	
ZAP1_P4_ Rv	CCACTGAAGTGCAAAGTGTGCG	

Fw - Forward primer; Rv - reverse primer. Restriction sites are underlined

* Primers used to measure the transcripts by qRT-PCR
